# Improving Quality of Care for Maternal and Newborn Health: Prospective Pilot Study of the WHO Safe Childbirth Checklist Program

**DOI:** 10.1371/journal.pone.0035151

**Published:** 2012-05-16

**Authors:** Jonathan M. Spector, Priya Agrawal, Bhala Kodkany, Stuart Lipsitz, Angela Lashoher, Gerald Dziekan, Rajiv Bahl, Mario Merialdi, Matthews Mathai, Claire Lemer, Atul Gawande

**Affiliations:** 1 Department of Health Policy and Management, Harvard School of Public Health, Boston, Massachusetts, United States of America; 2 Faculty of Epidemiology and Population Health, Infectious Disease Epidemiology Department, London School of Hygiene and Tropical Medicine, London, United Kingdom; 3 Women's and Children's Health Research Unit, Jawaharlal Nehru Medical College, Karnataka, India; 4 Center for Surgery and Public Health, Brigham and Women's Hospital, Boston, Massachusetts, United States of America; 5 Department of Patient Safety, World Health Organization, Geneva, Switzerland; 6 Department of Maternal, Newborn, Child and Adolescent Health, World Health Organization, Geneva, Switzerland; 7 Department of Reproductive Health and Research, World Health Organization, Geneva, Switzerland; 8 London Deanery, Barnet and Chase Farm Hospitals NHS Trust, Enfield, United Kingdom; The University of Adelaide, Australia

## Abstract

**Background:**

Most maternal deaths, intrapartum-related stillbirths, and newborn deaths in low income countries are preventable but simple, effective methods for improving safety in institutional births have not been devised. Checklist-based interventions aid management of complex or neglected tasks and have been shown to reduce harm in healthcare. We hypothesized that implementation of the WHO Safe Childbirth Checklist program, a novel childbirth safety program for institutional births incorporating a 29-item checklist, would increase delivery of essential childbirth practices linked with improved maternal and perinatal health outcomes.

**Methods and Findings:**

A pilot, pre-post-intervention study was conducted in a sub-district level birth center in Karnataka, India between July and December 2010. We prospectively observed health workers that attended to women and newborns during 499 consecutively enrolled birth events and compared these with observed practices during 795 consecutively enrolled birth events after the introduction of the WHO Safe Childbirth Checklist program. Twenty-nine essential practices that target the major causes of childbirth-related mortality, such as hand hygiene and uterotonic administration, were evaluated. The primary end point was the average rate of successful delivery of essential childbirth practices by health workers. Delivery of essential childbirth-related care practices at each birth event increased from an average of 10 of 29 practices at baseline (95%CI 9.4, 10.1) to an average of 25 of 29 practices afterwards (95%CI 24.6, 25.3; p<0.001). There was significant improvement in the delivery of 28 out of 29 individual practices. No adverse outcomes relating to the intervention occurred. Study limitations are the pre-post design, potential Hawthorne effect, and focus on processes of care versus health outcomes.

**Conclusions:**

Introduction of the WHO Safe Childbirth Checklist program markedly improved delivery of essential safety practices by health workers. Future study will determine if this program can be implemented at scale and improve health outcomes.

## Introduction

Reducing childbirth-associated mortality is a top global health priority but simple, effective methods to achieve it are severely lacking. Most of the 350,000 maternal deaths, 1·2 million intrapartum-related stillbirths, and 3·1 million neonatal deaths that occur each year could be avoided through the delivery of timely interventions proven to be effective and affordable [1], [2], [3], [4], [5]. Shifting place of delivery from home to hospital is a key strategy for improving childbirth outcomes and has led to unprecedented increases in institutional births in several countries [6], [7], [8]. But even as institutional birth rates rise, morbidity and mortality rates have been slow to fall [7], [9], [10]. Poor quality care in institutional births is recognized to be a major contributing factor to childbirth-related harm [11], [12], [13]. Improving facility-based care is a critical necessity yet no widely applicable, effective method currently exists.

In recent years checklist-based interventions have been adopted with increasing frequency in health to aid management of complex or neglected tasks that risk serious human harm. Integration of checklist programs into clinical practice has been shown to reduce deaths and complications in intensive care medicine and surgery [14], [15], [16], [17]. Several features of childbirth make a checklist-based strategy promising: the major causes of maternal and perinatal mortality are well described; most deaths occur within a narrow time window (twenty-four hours after birth); international guidelines for best practices exist but are not followed; and proven interventions are relatively inexpensive and easy to perform, but can be difficult to remember and execute in proper sequence [18], [19], [20].

In 2008, the World Health Organization (WHO) established a checklist-based childbirth safety program with the goal of determining whether a simple, low-cost, scalable intervention with potential for broader testing could be devised. A 29-item bedside WHO Safe Childbirth Checklist was developed according to previously established methodology and tested for usability in ten countries in Africa and Asia [21]. An implementation program was designed to maximize the likelihood of successful checklist adoption into clinical practice. We hypothesized that this program would increase the rate of delivery of essential childbirth practices linked with improved maternal, fetal, and neonatal health outcomes in a low income setting.

## Methods

### Study Design

We conducted a prospective, pre-post-intervention study observing childbirth practices of health workers at a sub-district level hospital in Karnataka, India. Our plans were to observe health workers attending to a minimum of 300 birth events before the intervention; introduce the checklist program; and then monitor health workers attending to a minimum of 300 birth events after the intervention. The total study was anticipated to last 6 months. The pilot hospital was selected on the basis of sufficient birth volume (minimum 250 births monthly), general availability of supplies, motivated leadership, and absence of other ongoing interventions. Basic emergency obstetric care and caesarean sections are offered at the facility [22]. Nurses provide care during most births. Other staff includes two obstetricians, one surgeon who performs caesarean sections, and an anesthetist. There are no pediatricians on staff. A co-investigator (BK) led the project locally and the hospital administration endorsed the intervention. Five data collectors were trained by the investigators to observe and document health worker practices. Data collectors were student nurses previously unknown to hospital staff with no clinical responsibilities. Inclusion criteria were health workers at the study site who cared for women and newborns from the time of admission for childbirth to discharge. Health workers providing care to mothers not involved in childbirth, including those being managed for abortions, miscarriages, and antenatal problems were excluded. Written informed consent was obtained from health workers and patients. The study was approved by ethics committees at JNMC Medical College in Karnataka, WHO, and the Harvard School of Public Health. The Indian Council of Medical Research also approved the study.

### Intervention

The intervention was a four-step checklist-based childbirth safety program designed and implemented using methods adapted from previous programs; in particular, CUSP and TeamSTEPPS [23], [24]. It involved (1) Engagement of local administrative and clinical leaders and identification of facility-based implementation leads; (2) Education about childbirth safety principles, deficiencies in current practice, and how to use the WHO Safe Childbirth Checklist during a one-day learning session; (3) Execution beginning with one week of simulation and supervised practice; and (4) Evaluation and ongoing monitoring [25].

The hospital-based implementation leads (an administrator, a senior physician, and a senior labor nurse) were selected by the facility and trained by the investigators. They introduced the checklist program to staff during the one-day learning session and monitored its ongoing use. Learning was supported by written materials, lectures, an instructional video, and hands-on simulation. Following introduction of the program, the implementation leads supervised use of the checklist and offered strategies for improvement in performance and learning when their full-time clinical schedules allowed. An outside physician “coach” visited the facility fortnightly to provide additional support to help staff improve adherence to the checklist program.

The WHO Safe Childbirth Checklist consists of succinct reminders of essential steps for safe childbirth care (see Table S1). Items on the checklist address the major causes of maternal deaths (*i.e.*, hemorrhage, infection, obstructed labor, and hypertensive disorders), intrapartum-related stillbirths (*i.e.*, inadequate intrapartum care), and neonatal deaths (*i.e.*, intrapartum-related events, infection, and complications of prematurity) in lower income countries [18], [26], [27], [28]. Checklist items are organized for use at four critical junctures in care during birth: at the time the woman is admitted, when the woman begins to push or before cesarean, within one hour after birth, and before discharge. Modifications to the checklist are encouraged to align content with local practice. Adjustments made by the pilot site's review committee were the following: change from “does mother need referral” to “does mother need review by obstetrician,” minor change to maternal antibiotic administration criteria (specifying labor >24 hours in a primigravida or labor >12 hours in multipara as indications), minor change to maternal magnesium sulfate administration criteria (specifying diastolic blood pressure threshold at 100 mmHg instead of 110 mmHg), inclusion of ASHA (Accredited Social Health Activist) workers as satisfactory birth companions, requirement that nurses introduce themselves by name to laboring women, removal of the newborn special care and monitoring item (all ill newborns were referred to other facilities since there was no pediatrician on staff), and removal of the follow-up after discharge item (the existing follow-up process was felt by local staff to be sufficient). No additional equipment, supplies, or medications were provided. Checklist use was not mandatory; individual health workers could decide for themselves whether or not to use the checklist during any given patient encounter. Completed checklists were attached to the mothers' charts.

### Data Collection

Data were collected through observation of health workers' practices and review of birth registers. Observation data were recorded on standardized data sheets by data collectors who directly observed health workers. Observation took place 24 hours daily for a minimum of six days weekly; unobserved days were selected at random. Practical limitations precluded continuous observation of each woman from the time of admission until discharge. Observation therefore took place at three specific periods: on admission, continuously from the time of pushing until one hour after delivery, and before discharge. Checklist use was observed during the post-intervention period. Data collectors did not interact with patients or health workers during their observations. For ethical reasons, they were instructed to notify health workers if they observed a potentially harmful condition or practice. Data quality was assured through periodic assessment of data collector skills (confirming they achieved 100% concordance on a sample of three observations), parallel observations by the local study coordinator once weekly, and on-site review of all observation forms within 72 hours. The data management system had range, plausibility, and cross-validation checks confirming all data were logical. Double data entry was performed for a sample of the observation forms.

### Outcomes

The primary outcome was the average rate of successful delivery of essential childbirth practices by health workers at each birth event. A birth event was defined as the period from admission for childbirth to discharge. Twenty-nine practices were evaluated. Successful delivery of individual practices was defined by completion of a predetermined set of process indicators (see Table S2). For several practices this required proper assessment of the mother or baby, recognition of abnormal signs or symptoms, and execution of appropriate action. Essential childbirth practices relating to maternal care were referral when indicated, partograph use, periodic assessment of infection risk and antibiotic administration when indicated, periodic assessment of hypertensive-disease risk and magnesium sulfate administration when indicated, assessment of HIV risk and anti-retroviral administration when indicated, presence of a birth companion, good hand hygiene, periodic danger sign counseling, presence of an assistant for birth, oxytocin administration within 1 minute after birth, periodic blood loss estimation and bleeding risk, and family planning discussion. Essential childbirth practices relating to newborn care were use of a sterile blade to cut the umbilical cord, proper thermal and resuscitation care, referral when indicated, assessment of HIV risk and anti-retroviral administration when indicated, breastfeeding within one hour, periodic assessment of infection risk and antibiotic administration when indicated, assessment of adequate feeding before discharge, and periodic danger sign counseling.

Observed rates of in-facility maternal deaths, newborn deaths, and stillbirths were analyzed as secondary outcomes. The frequency of medication administration before and after the intervention was also measured.

### Statistical Analysis

We aimed to collect data on a minimum of 300 consecutively enrolled birth events during each phase of the study. The sample size was calculated to detect a 20% absolute increase in the average rate of essential childbirth practices successfully delivered by health workers after introduction of the checklist program, with a statistical power of 80% and an alpha value of 0.05, using a generalized estimating equations (GEE) test accounting for clustering by provider [29], [30].

Each of the 29 individual childbirth practices were quantified in terms of the proportion successfully delivered, and GEE methods were again used to adjust confidence intervals to account for clustering by provider. Rao-Scott Chi-Square tests were used to test whether possible categorical confounders (categorical patient characteristics) had different distributions before and after the intervention, accounting for clustering by provider. If covariates were found to be imbalanced over the two phases, regression analyses were conducted to adjust for possible confounding due to patient characteristics.

Using the Bonferroni approach to adjust the overall 5% Type I Error rate for the 30 before versus after comparisons (each of the 29 individual childbirth practices plus the average rate of practices delivered), a P-value less than 0.05/30 = 0.0017 would be declared significant.

Our secondary outcome was the change in observed rates of in-facility maternal deaths, newborn deaths, and stillbirths, although the study was not powered to detect a difference in mortality. Secondary outcomes were exploratory and the Type I error rate was not adjusted.

Analyses were performed with SAS version 9·2 (SAS Institute Inc, Cary, North Carolina).

## Results

We observed health workers attending to 499 birth events during the baseline period (July–September, 2010) and 795 birth events after introduction of the checklist program (September–December, 2010). All hospital staff involved in childbirth were invited to participate and agreed to do so, and there was no staff turnover during the study. The pre- and post-intervention periods did not overlap and data was not collected during the brief period when the program was introduced. Table 1 lists the number of observations made at each period in the flow of care. Demographic characteristics of women and newborns are shown in Table 2; there were no differences in the two phases of the study. The checklist was observed to be used by health workers at least 95% of the time at each of the four checklist pause points in the post-intervention period.

**Table 1:** Number of childbirth events observed at each period before and after intervention.

Variable	Before	After
**Total number of childbirth events at GH**	**624**	**889**
**Total number of childbirth events observed**	**499**	**795**
Admissions observed	405	638
Deliveries and immediate postnatal periods observed	388	583
Discharges observed	338	489

**Table 2:** Demographic characteristics of women and newborns before and after intervention.

Characteristic		Before (n = 499)	After (n = 795)
Age (yrs)		23+/−3	23+/−3
Parity (%)	0	44	48
	1–3	54	50
	>4	2	1
Referred case (%)^a^		4	2
Unbooked case (%)^b^		73	69
Previous caesarean section (%)		7	6
Sex of newborn (%)	Male	51	52
	Female	49	49
Birth weight (%)	<1500 g	5	5
	1500 g–2500 g	13	12
	>2500 g	82	83
Multiple birth (%)		1	1

a Referred to study facility from another facility after labor started.

b Attended fewer than 3 antenatal appointments.

**Figure 1:**
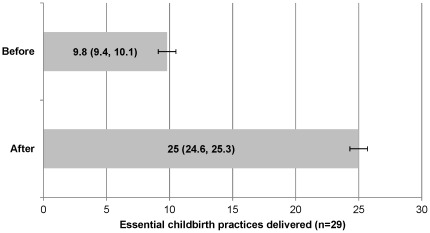
Average rate of successful delivery of essential childbirth practices before and after intervention (p<0.001).

**Figure 2:**
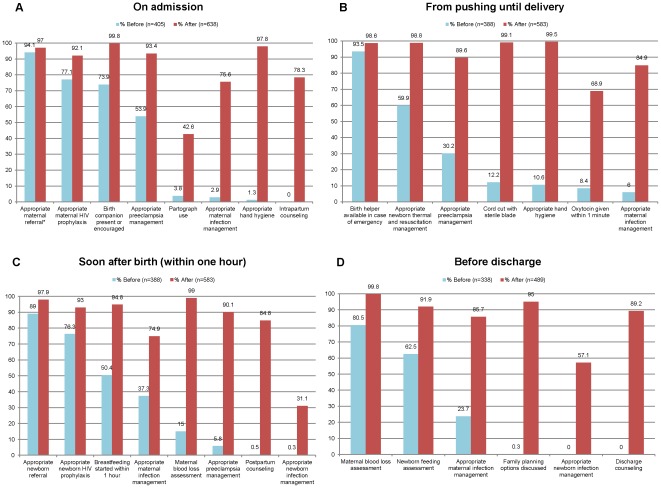
Changes in rates of delivery of specific childbirth practices before and after intervention; (2a) On admission; (2b) From pushing until delivery; (2c) Soon after birth (within one hour); (2d); Before discharge (*P value = 0.052; all others p≤0.001).

The rate of successful delivery of essential practices at each birth event increased from an average of 10 of 29 practices at baseline (95%CI 9.4, 10.1) to an average of 25 of 29 practices afterwards (95%CI 24.6, 25.3; p<0.001)(Figure 1). The Bonferroni correction did not affect the outcomes that were declared significant since all P-values less than 0.05 were also less than 0.0017. Figure 2 shows the rates of successful completion of individual practices before and after introduction of the checklist program. There was significant improvement in the delivery of every practice except maternal referral.

**Figure 3:**
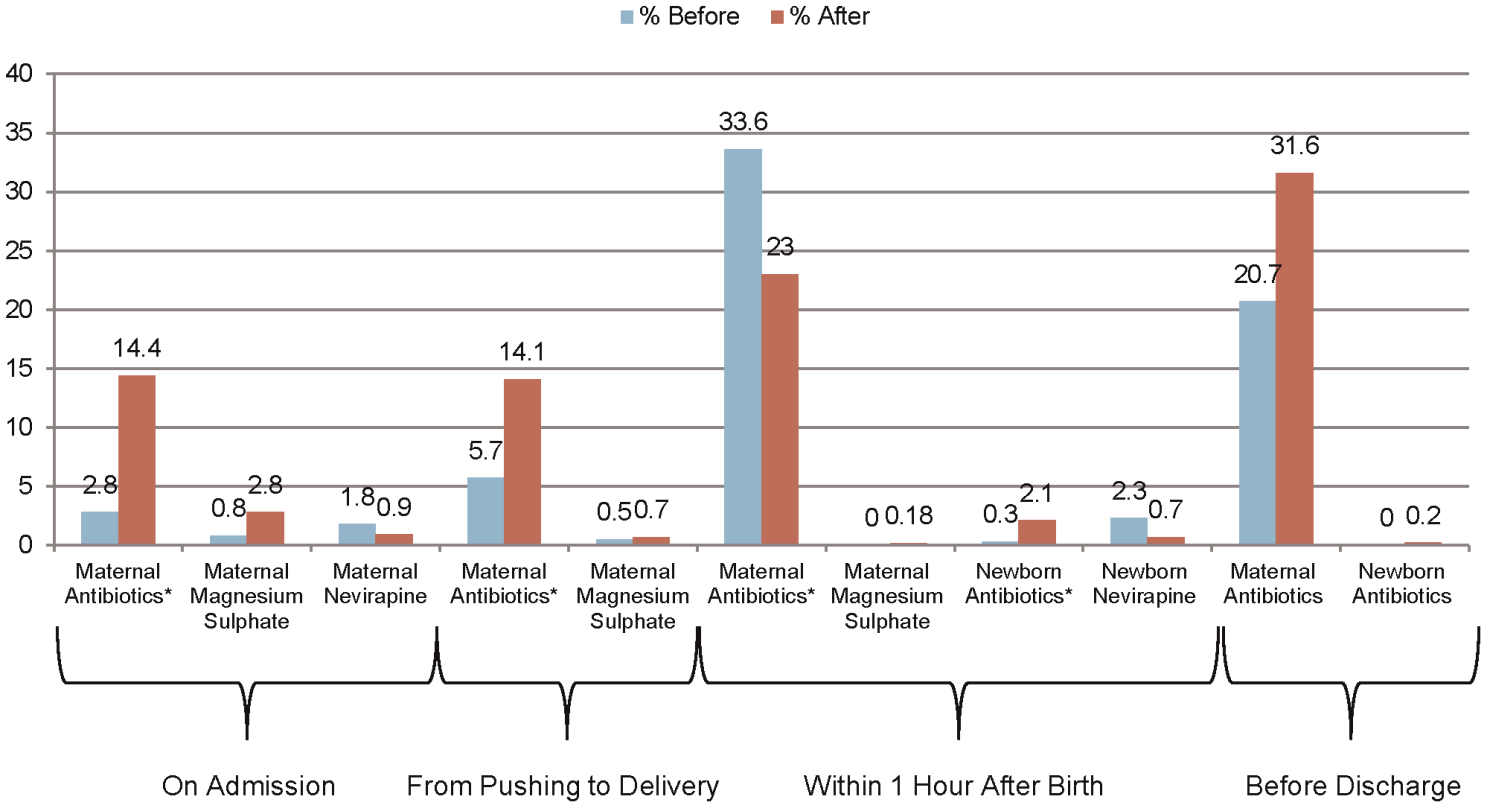
Frequency of initiation of medication therapy before and after intervention (*p<0.05).

Figure 3 shows the frequency of initiation of medication therapy at each period of care. The use of medications increased at some periods and decreased at others. There were no differences in observed maternal and neonatal deaths in the two study periods, though the study was not powered to detect significant differences in mortality (Table 3). The stillbirth rate showed a declining trend after introduction of the checklist program.

**Table 3:** Observed in-facility mortality before and after intervention.

Variable	Before	After	*p*-value
Maternal deaths per 100,000 observed women (n/N)	203 (1/492)	126 (1/791)	0.87
Neonatal deaths per 1,000 observed discharges (n/N)	5.9 (2/337)	6.1 (3/489)	0.99
Total stillbirths per 1,000 observed deliveries (n/N)	33.6 (13/387)	15.5 (9/582)	0.07

## Discussion

In this pilot study, a novel checklist-based childbirth safety program led to a marked increase in delivery of essential childbirth practices linked with improved maternal, fetal, and newborn outcomes. Overall, there was an average 150% increase in adherence to accepted clinical practices at any given birth event, and 28 of 29 individual practices were delivered with significantly greater frequency. Strategies for achieving quality improvements in institutional births in low income settings are lacking. These results suggest the potential for this approach to improve maternal and perinatal care.

While these results were found in just a single hospital, research in this area has not previously demonstrated interventions that are comprehensive (to capture sufficient behaviors to have a chance of saving lives) and simple (to be scalable and sustainable). Moreover, facilitating behavior change to increase adherence to evidence-based healthcare practices is known to be challenging [31], [32]. In this initial work we aimed to prove that such methods could be devised and produce measurable change.

The ways by which a checklist-based approach improves care during childbirth warrants exploration to better understand how this intervention produced such promising results. We believe the intervention had three primary mechanisms of effect: (1) as a checklist instrument that reinforced for health workers a core set of essential practices that must be completed at each and every birth; (2) as a reminder to complete these practices at the most crucial period – at the bedside at the moment of care; and (3) as a tool that highlighted gaps in the existing system of care at the facility which enabled local staff to take steps to effectively strengthen their own health system to ensure adherence to checklist practices. No additional investments in equipment or supplies were made, and no incentives were given. The local team seemed inspired by the checklist program and developed a personal interest in helping it to succeed.

There were changes in behavioral patterns that were individual in nature, for example improvement in health workers washing their hands with soap and water and wearing clean gloves (by comparison, before the intervention soap was not used routinely). Other improvements resulted from system changes. For instance, after introduction of the program it became apparent that no structure was in place to adequately monitor women and newborns immediately after delivery, which brought to light the difficulty in reliably completing crucial assessments and practices at that time. In response, the local staff took the initiative to convert an underutilized room adjacent to the labor ward into a postpartum bay where women and newborns were observed for at least one hour after delivery. The checklist program also helped to identify similar deficiencies in the discharge process, which was subsequently systematized by the local staff. The program reinforced the importance of ensuring that medicines and supplies were readily available to health workers on the labor ward and not kept remotely in the hospital store. Finally, the intervention appeared to improve communication and teamwork. Nursing staff used the checklist as a framework for communicating patient information at shift changes as well as conducting daily safety rounds to discuss complicated cases as a team.

Medication use, which increased in some periods and decreased in others, was a partial contributor to improved practices. Improved assessment of mothers and newborns was often the most significant factor in health workers' successful adherence to essential practices. We suspect that increased medication usage reflected improved awareness of appropriate indications for administration (for instance, increased antibiotic administration to at-risk newborns in the postnatal period) and that decreased medication administration reflected a decline in overuse practices (for instance, a reduction in the traditional practice of administering antibiotics after birth for all episiotomies).

We recognize that a simple paper checklist alone is unlikely to result in lasting behavior change [33]. In this study the WHO Safe Childbirth Checklist was the central component of an implementation program based on a well-described change model carried out by hospital administration and clinical leaders [23]. The program involves engaging and empowering the local team; providing education on best practices and existing deficiencies; discussing potential barriers and introducing the checklist through focused training; and establishing a mechanism for ongoing monitoring and evaluation [34]. This is a comprehensive behavior change strategy facilitated by a checklist program. We found that this approach was associated with rapid uptake by the local team and low implementation costs.

This study has several limitations. A risk of the pre-post-intervention design is confounding by secular trends. The study period was, however, limited to six months and no difference was observed in the characteristics of the women and newborns in the two phases of the study. No other interventions took place during the study period and there were no changes in hospital staffing. For these reasons, secular trends alone were unlikely to be responsible for the observed differences.

A potential Hawthorne effect, in which subjects' behavior is influenced by an awareness of being observed, is another methodological limitation given that independent observers conducted the evaluation of health workers' performance [35]. This methodology was selected for its obvious advantages over self-reporting and for practicality (for instance, video cameras in this setting would have been infeasible). We worked to minimize Hawthorne effect by employing the same data collectors before and after the intervention, by having the health workers observed in the same way for both periods of observation, and by structuring nearly continuous periods of observation from the start of the study so that health workers had the opportunity to become accustomed to the presence of observers.

Additional concerns relate to the study's generalizability and sustainability. The pilot facility is representative of first-level referral centers in India albeit with a motivated local team and general accessibility to childbirth equipment and supplies. These factors undoubtedly contributed to implementation success. The potential efficacy of this approach in other settings is unclear and merits further study. Regarding sustainability, there is evidence from other disciplines to suggest that checklist programs remain to be associated with sustained health improvements and positive attitudes toward the programs up to at least 18 months after the initial introduction and evaluation periods [36], [37], [38]. A follow-up to this pilot study is now being organized to assess checklist use and adherence to essential practices more than 12 months after the initial investigation.

Lastly, this pilot study focused on processes of care as indicators of quality. Though the observed stillbirth rates showed a declining trend, the sample size in this pilot investigation was insufficient to detect significant differences in mortality. A multi-center study in north India is currently underway to measure the impact of the program on a composite measure of severe maternal, fetal, and newborn harm. Enrollment for this prospective randomized trial is anticipated to begin in late 2012.

### Conclusion

Most maternal and newborn deaths, and many stillbirths, are avoidable. Assuring the delivery of key evidence-based interventions during childbirth is critical to optimizing care for women and newborns and helping priority countries to achieve progress toward Millennium Development Goals Four and Five. In this study, implementation of a novel checklist-based childbirth safety program led to improved quality of care delivered by health workers attending to institutional deliveries. Future study is required to determine whether a checklist-based approach to promoting safety in childbirth also reduces harm and saves lives.

## Supporting Information

Table S1 **Elements of the WHO Safe Childbirth Checklist.**
(DOCX)

Table S2 **Definitions of childbirth practices used in the WHO Safe Childbirth Checklist program pilot study.**
(DOC)
