# Supplementary material for: Improving Quality of Care for Maternal and Newborn Health: Prospective Pilot Study of the WHO Safe Childbirth Checklist Program
Source: PLoS One. 2012 May 16;7(5):e35151. doi: 10.1371/journal.pone.0035151 (PMC3353951; doi:10.1371/journal.pone.0035151)
Supplement: Table S1 — Elements of the WHO Safe Childbirth Checklist. (DOCX) [file pone.0035151.s001.docx]

**Table S1. Elements of the WHO Safe Childbirth Checklist**

| **Checklist Item** |  | **Qualifying Caption** |
| --- | --- | --- |
| ***On admission of the mother to the birth facility*** |  |  |
| Does mother need referral? | □ Yes, organized | According to facility's criteria |
|  | □ No |  |
| Partograph started? | □ Yes | Start plotting when cervix ≥ 4 cm, then cervix should dilate ≥ 1 cm/hr. Every 30 min: plot heart rate, contractions, fetal heart rate. Every 2 hours: plot temperature. Every 4 hours: plot blood pressure |
|  | □ No, will start when ≥ 4 cm |  |
| Does mother need to start antibiotics? | □ Yes, given | Give if temperature > 38^o^C, foul-smelling vaginal discharge, rupture of membranes >18 hours, OR labor >24 hours |
|  | □ No |  |
| Does mother need to start magnesium sulfate? | □ Yes, given | Give if (1) diastolic blood pressure ≥110 mmHg and 3+ proteinuria, OR (2) diastolic blood pressure ≥90 mmHg, 2+ proteinuria, and any: severe headache, visual disturbance, OR epigastric pain |
|  | □ No |  |
| Does mother need to start anti-retroviral medicine? | □ Yes, given | Give if mother is HIV+ and in labor |
|  | □ No |  |
| □ Supplies available to clean hands and wear gloves for each vaginal exam |  |  |
| □ Birth companion encouraged to be present at birth |  |  |
| □ Confirm that mother/companion will call for help during labor if mother has a danger sign |  | Call for help if bleeding, severe abdominal pain, severe headache, visual disturbance, urge to push, OR difficulty emptying bladder |
| ***Just before pushing (or before Cesarean)*** |  |  |
| Does mother need to start antibiotics? | □ Yes, given | Give if temperature > 38^o^C, foul-smelling vaginal discharge, rupture of membranes >18 hours now, labor >24 hours now, OR cesarean section |
|  | □ No |  |
| Does mother need to start magnesium sulfate? | □ Yes, given | Give if (1) diastolic blood pressure ≥110 mmHg and 3+ proteinuria, OR (2) diastolic blood pressure ≥90 mmHg, 2+ proteinuria, and any: severe headache, visual disturbance, OR epigastric pain |
|  | □ No |  |
| Are essential supplies at bedside for mother? | □ Gloves | Prepare to care for mother immediately after birth: (1) Exclude 2^nd^ baby, (2) Give oxytocin within 1 minute, (3) Controlled cord traction to deliver placenta, (4) Massage uterus after placenta is delivered |
|  | □ Soap and clean water |  |
|  | □ Oxytocin 10 IU in syringe |  |
| Are essential supplies at bedside for baby? | □ Clean towel | Prepare to care for baby immediately after birth: (1) Dry baby and keep warm, (2) If not breathing: stimulate and clear airway, (3) If still not breathing: cut cord, ventilate with bag-and-mask, (4) shout for help |
|  | □ Sterile blade to cut cord |  |
|  | □ Suction device |  |
|  | □ Bag-and-mask |  |
| □ Assistant identified and informed to be ready to help at birth if needed? |  |  |
| ***Soon after birth (within 1 hour)*** |  |  |
| Is mother bleeding too much? | □ Yes, shout for help | If bleeding >500 ml, or if >250 ml and severely anemic: massage uterus, consider additional uterotonic, start intravenous line, treat cause |
|  | □ No |  |
| Does mother need to start antibiotics? | □ Yes, given | Give if placenta manually removed, or if temperature >38^o^C and any: foul-smelling vaginal discharge. lower abdominal tenderness, rupture of membranes >18 hours at time of delivery, OR labor >24 hours at time of delivery |
|  | □ No |  |
| Does mother need to start magnesium sulfate? | □ Yes, given | Give if (1) diastolic blood pressure ≥110 mmHg and 3+ proteinuria, OR (2) diastolic blood pressure ≥90 mmHg, 2+ proteinuria, and any: severe headache, visual disturbance, OR epigastric pain |
|  | □ No |  |
| Does baby need referral? | □ Yes, organized | According to facility's criteria |
|  | □ No |  |
| Does baby need to start antibiotics? | □ Yes, given | Give if antibiotics were given to mother, or if baby has any: breathing too fast (>60 breaths/min) or too slow (<30 breaths/min), chest in-drawing, grunting, convulsions, no movement on stimulation, OR too cold (temperature <35^o^C and not rising after warming) or too hot (temperature >38^o^C) |
|  | □ No |  |
| □ Does baby need special care and monitoring? |  | Recommended if more than 1 month early, birth weight <2500 grams, needs antibiotics, OR required resuscitation |
| Does baby need to start an anti-retroviral medicine? | □ Yes, given | Give anti-retroviral medicine if mother is HIV+ |
|  | □ No |  |
| □ Started breastfeeding and skin-to-skin contact? (if mother and baby are well) |  |  |
| □ Confirm that mother/companion will call for help if: |  | Mother has bleeding, severe abdominal pain, severe headache, visual disturbance, breathing difficulty, fever/chills, OR difficulty emptying bladder |
|  |  | Baby has fast or difficulty breathing, fever, unusually cold, stops feeding well, less activity than normal, OR whole body becomes yellow |
| ***Before discharge*** |  |  |
| Is mother’s bleeding controlled? | □ Yes |  |
|  | □ No, treat and delay discharge |  |
| Does mother need to start antibiotics? | □ Yes, given | Give if temperature >38^o^C and any: chills, foul-smelling vaginal discharge, OR lower abdominal tenderness |
|  | □ No |  |
| Does baby need to start antibiotics? | □ Yes, give antibiotics, delay discharge, and give special care or refer | Give if breathing too fast (>60 breaths/min) or too slow (<30 breaths/min), chest in-drawing, grunting, convulsions, no movement on stimulation, too cold (temperature <35^o^C and not rising after warming) or too hot (temperature >38^o^C), stopped breastfeeding well, OR umbilical redness extending to skin or draining pus |
|  | □ No |  |
| Is baby feeding well? | □ Yes |  |
|  | □ No, help and delay discharge |  |
| □ Family planning options discussed and offered to mother |  |  |
| □ Confirm that mother/companion will call for help after discharge if: |  | Mother has bleeding, severe abdominal pain, severe headache, visual disturbance, breathing difficulty, fever/chills, OR difficulty emptying bladder |
|  |  | Baby has fast or difficulty breathing, fever, unusually cold, stops feeding well, less activity than normal, OR whole body becomes yellow |
| □ Follow-up arranged for mother and baby |  |  |
