# Supplementary material for: Improving Quality of Care for Maternal and Newborn Health: Prospective Pilot Study of the WHO Safe Childbirth Checklist Program
Source: PLoS One. 2012 May 16;7(5):e35151. doi: 10.1371/journal.pone.0035151 (PMC3353951; doi:10.1371/journal.pone.0035151)
Supplement: Table S2 — Definitions of childbirth practices used in the WHO Safe Childbirth Checklist program pilot study. (DOC) [file pone.0035151.s002.doc]

**Table S2: Definitions of childbirth practices used in the WHO Safe Childbirth Checklist program pilot study**

| **Childbirth practice** | **Definition** |
| --- | --- |
| ***On admission*** |  |
| Appropriate maternal referral | Mother assessed for predetermined referral criteria (hemogloblin <7 g/dL and in labor or suffering from refractory eclampsia) and referred within one hour after admission if indicated |
| Appropriate maternal HIV prophylaxis | Maternal HIV status checked and nevirapine given to mother within one hour after admission if mother is HIV positive |
| Birth companion present or encouraged | Family member or community health worker present with mother or encouraged to be present if not present |
| Appropriate preeclampsia management | Mother assessed for signs and symptoms of preeclampsia (diastolic blood pressure >100 mmHg, or diastolic blood pressure >90 mmHg and severe headache or visual disturbance or epigastric pain) and treated with magnesium sulfate within one hour after admission if indicated |
| Partograph use | Partograph labeled with mothers name and admission vital signs within one hour after admission; if in active labor, then partograph charting initiated within one hour after admission |
| Appropriate maternal infection management | Mother assessed for predetermined risk factors and signs of infection (temperature >38^o^C/100.4^o^F or foul-smelling vaginal discharge or rupture of membranes at home or labor >24 hours in primigravida or labor >12 hours in multipara) and treated with antibiotics within one hour after admission if indicated |
| Appropriate hand hygiene | Hands washed with clean water and soap, and clean gloves worn for admission vaginal examination |
| Intrapartum counseling | Mother and/or birth companion verbally informed within one hour after admission about danger signs for which they should call for help during labor: bleeding, severe abdominal pain, severe headache, visual disturbance, cannot empty bladder every 2 hours, or urge to push |
| ***From the time of pushing until delivery*** |  |
| Birth helper available in case of emergency | Assistant identified and informed to be available to assist at delivery in event of complications |
| Appropriate newborn thermal and resuscitation management | Newborn assessed and given appropriate immediate newborn care (newborn dried and kept warm, if not breathing then airway cleared and newborn stimulated, and if still not breathing then newborn ventilated with bag-and-mask) |
| Appropriate preeclampsia management | Mother assessed for signs and symptoms of preeclampsia (diastolic blood pressure >100 mmHg, or diastolic blood pressure >90 mmHg and severe headache or visual disturbance or epigastric pain) and treated with magnesium sulfate before delivery if indicated |
| Cord cut with sterile blade | Sterile blade used to cut umbilical cord after birth |
| Appropriate hand hygiene | Hands washed with clean water and soap, and clean gloves worn for delivery |
| Oxytocin given within 1 minute | Oxytocin 10 IU by intramuscular injection administered to mother within one minute after delivery |
| Appropriate maternal infection management | Mother assessed for predetermined risk factors and signs of infection (temperature >38^o^C/100.4^o^F or labor now >24 hours in primigravida or labor now >12 hours in multipara) and treated with antibiotics before delivery if indicated, or antibiotics administered if proceeding to caesarean section |
| ***Soon after birth (within one hour)*** |  |
| Appropriate newborn referral | Newborn assessed for predetermined referral criteria (birth weight <2 kilograms or gestation <32 weeks or having breathing difficulty if required bag-and-mask) and referred within one hour after delivery if indicated |
| Appropriate newborn HIV prophylaxis | Maternal HIV status checked and nevirapine given to newborn within one hour after delivery if indicated |
| Breastfeeding started within 1 hour | Breastfeeding initiated within one hour after delivery |
| Appropriate maternal infection management | Mother assessed for predetermined signs of infection (if placenta manually removed or if temperature >38^o^C/100.4^o^F and labor was >24 hours for primigravada or >12 hours for multipara) and treated with antibiotics within one hour after delivery if indicated |
| Maternal blood loss assessment | Mother's postpartum blood loss checked within one hour after delivery |
| Appropriate preeclampsia management | Mother assessed for signs and symptoms of preeclampsia (diastolic blood pressure >100 mmHg, or diastolic blood pressure >90 mmHg and severe headache or visual disturbance or epigastric pain) and treated with magnesium sulfate within one hour after delivery if indicated |
| Pospartum counseling | Mother and/or birth companion verbally informed within one hour after delivery about danger signs in mother (bleeding, severe abdominal pain, severe headache, visual disturbance, breathing difficulty, fever or chills, difficulty emptying bladder) and newborn (fast or difficulty breathing, fever, unusually cold, stops feeding well, less activity than normal, whole body becomes yellow) for which they should call for help after delivery |
| Appropriate newborn infection management | Newborn assessed for predetermined signs of infection (mother given antibiotics or gestation < 32 weeks or birth weight <1.5 kilograms or breathing rate >60 breaths/minute or breathing rate <30 breaths/minute or convulsions or floppy or temperature < 35^o^C/95^o^F despite warming measures or >38^o^C/100.4^o^F) and treated with antibiotics within one hour after delivery if indicated |
| ***Before discharge*** |  |
| Maternal blood loss assessment | Mother's postpartum blood loss checked before discharge |
| Newborn feeding assessment | Feeding status of newborn checked before discharge |
| Appropriate maternal infection management | Mother assessed for predetermined signs of infection (temperature >38^o^C/100.4^o^F and either foul-smelling vaginal discharge or lower abdominal tenderness) and treated with antibiotics before discharge if indicated |
| Family planning options discussed | Importance of family planning and two or more available options discussed before discharge |
| Appropriate newborn infection management | Newborn assessed for predetermined signs of infection (newborn breathing rate >60 breaths/minute or <30 breaths/minute or convulsions or floppy or newborn temperature <35^o^C/95^o^F despite warming measures or >38^o^C/100.4^o^F) and treated with antibiotics before discharge if indicated |
| Discharge counseling | Mother and/or birth companion verbally informed before discharge about danger signs in mother (bleeding, severe abdominal pain, severe headache, visual disturbance, breathing difficulty, fever or chills, difficulty emptying bladder) and newborn (fast or difficulty breathing, fever, unusually cold, stops feeding well, less activity than normal, whole body becomes yellow) for which they should seek care after discharge |
